# L1 grammatical attrition through the acquisition of competing L2 discourse features

**DOI:** 10.3389/fpsyg.2024.1399870

**Published:** 2024-09-13

**Authors:** Liz Smeets

**Affiliations:** Department of Languages, Literatures and Linguistics, York University, Toronto, ON, Canada

**Keywords:** clitic left dislocation, L1 attrition, L2 transfer, Italian, Romanian, discourse-syntax interface

## Abstract

A question in language acquisition research is whether attrition can affect L1 grammatical representation, and if so, under what conditions. This paper tests the Attrition via Acquisition (AvA) model, which takes a Feature Reassembly approach to predict how, in case on high degrees of similarity between the L1 and L2, the acquisition of L2 discourse-driven morpho-syntactic properties may affect L1 feature representations after a prolonged change in the speaker’s primary linguistic input during adulthood. As a test case, we use the different features (*specificity* versus *discourse anaphoricity*) associated with Clitic Left Dislocation (CLLD) in Romanian and Italian, examining the grammars of Romanian first-generation immigrants with either L2 Italian or L2 English (a language without CLLD). Using a context-dependent Acceptability Judgment task and a Written Elicitation task we found evidence for L2-induced grammatical attrition, resulting in the addition of an L2 option without the loss of an L1 option, as predicted by the AvA. Attrition was found for participants who immigrated during adolescence or early adulthood and who are more likely to consider Italian their most proficient and most used language. We compare our findings on attrited L1 grammars to the results of a recent study reporting on near-native L2 Italian and L2 Romanian grammars by Romanian and Italian native speakers. Our findings contribute to an increasing body of literature showing that L1 attriters and L2 learners can end up with very similar grammars and confirm the importance of studying second language acquisition and L1 loss within a broader picture of bilingual development.

## 1 Introduction

The current study focuses on the potential modification or restructuring of a speaker’s L1 grammatical representation (‘grammatical attrition’) in individuals whose primary linguistic input has changed for a prolonged period of time due to immigration to a country where a different language is spoken. The available empirical evidence on grammatical attrition largely supports the idea that effects of attrition are relatively minor in late bilinguals compared to early bilinguals such as heritage speakers; an overview by Montrul (2008) reports that studies on adult L1 attrition of morpho-syntactic phenomena rarely ever find that attriters make morpho-syntactic errors in more than 5% of the contexts in which a specific morpheme is required. Neurolinguistic research also supports the idea that potential attritters behave like native speakers on morpho-syntactic violations (Bergmann et al., 2015; but see Kasparian, 2015 for arguments against this claim). Despite the observation that L1 attrition of morpho-syntactic phenomena is rare in speakers who acquired their L2 in adulthood, such cases have been reported (e.g., Sorace, 1993; Gürel, 2002; Iverson, 2012).

However, we still know quite little about what properties can undergo attrition and under what linguistic and extralinguistic conditions structural changes to L1 grammars can occur. To address this question as well as the rarity of L1 attrition, Hicks and Domínguez (2020) recently proposed the Attrition via Acquisition (AvA) model, a formal model of grammatical attrition which presents testable predictions for how changes to the L1 grammar may occur and uses principles of generative grammar in combination with psycholinguistic approaches on language acquisition to account for the rare occurrence of grammatical attrition.

The current study tests the predictions of the AvA on potential changes to L1 grammars in first generation immigrants who learned the L2 as adults and are living in the L2 environment. We focus on a phenomenon at the syntax-discourse interface, specifically the use of Clitic Left Dislocation (CLLD) in Romanian and its differences from Italian CLLD. To date, most previous studies on L1 attrition of discourse-syntactic phenomena have focused on the interpretation of anaphoric forms, like null and overt pronouns in null subject languages (Gürel, 2002; Tsimpli et al., 2004; Gürel and Yılmaz, 2011; Domínguez, 2013; Kaltsa et al., 2015; Chamorro et al., 2016; Miličević and Kraš, 2017) or pronominal and demonstrative pronouns in German (Wilson, 2009; Wilson et al., 2009), but attrition in CLLD has not previously been tested. To disentangle effects of L2 transfer on attrition from general L1 disuse we compare two groups of speakers with different L2s: one group whose L2 has CLLD (Italian), but uses this construction in different discourse contexts than Romanian, and a group of speakers whose L2 does not have CLLD (English). According to the AvA, grammatical attrition is only expected for properties where analogous forms exist in the L1 and the L2 but where these forms differ in their behavior due to differences in their feature specifications, as is the case for Romanians who acquired Italian as an L2. We furthermore examine the effects of language-external factors, such as age of immigration (including participants who immigrated during and after adolescence), relative L1 and L2 use, and L2 proficiency as factors contributing to attrition. Results from the current study are furthermore compared to those of an earlier study on L2 acquisition reported in Smeets (2023), as we observed interesting patterns of crosslinguistic influence in L2 acquisition and in L1 attrition that are alike.

## 2 Reduced processing efficiency or changes in grammatical representation?

Most previous attrition studies investigating linguistic phenomena involving the integration of discourse information into the syntax have been conducted in light of the Interface Hypothesis (IH; Sorace, 2011). The IH predicts more optionality and variability in the performance of attriters compared to non-attriters for structures that require the integration of linguistic and non-linguistic information, like discourse information (Tsimpli et al., 2004; Sorace, 2011). Differences between groups are particularly predicted to be found in real-time processing, arguably due to reductions in working memory and processing efficiency (Rothman and Slabakova, 2011). Note that the IH is a theory of processing and assumes that attriters who migrated in adulthood do not have a grammar that qualitatively differs from monolingual non-attriters who speak the same L1. Instead, the grammatical errors that L1 attriters make are argued to be due to a momentary conflict between their two linguistic systems, causing instances of disfluency and optionality in the use of morpho-syntactic properties. Processing approaches to attrition more generally have argued that a lower frequency of activation can cause processing delays in bilinguals independently of L1-L2 differences (Gollan et al., 2005) or that it is cross-linguistic transfer in the form of competition and spread of activation from the L2 or other languages that can lead to less efficient processing (Marian and Spivey, 2003; Blumenfeld and Marian, 2007).

To examine whether attrition is due to a momentary conflict between the two grammars or a different grammatical representation, Chamorro et al. (2016) compared three groups of native Spanish speakers, two of which had been living in the United Kingdom for at least 5 years, and a group of Spanish native controls who had recently moved to the United Kingdom with very little knowledge of English. The two experimental groups differed in that the speakers in one group were recently re-exposed to Spanish only. Participants were tested on anaphora resolution of null and overt pronouns in Spanish using sentences like (1) where null pronouns (*pro*) have been shown to prefer subject antecedents while overt pronouns favor object antecedents. Two tasks were used, an online eye-tracking task and an untimed naturalness judgment task, as the authors assumed that online tasks measure real-time language processing and untimed offline tasks reflect knowledge representation.

1. La  madre   saludó   a   la  chica  cuando   ella/ *pro* cruzaba una calle  con mucho tráfico.
  The mother  greeted  to   the girl    when    she/*pro*  crossed a   street with much  traffic.
  ‘The mother greeted the girl when she crossed a street with lots of traffic.’
  Adapted from Chamorro et al. (2016, Ex. 8).

The results revealed that the monolingual and the re-exposed groups had faster go-past times in the critical region (pronoun or *pro*) when the overt pronoun had an object antecedent and when the null pronoun had a subject antecedent. The attrited group was faster when the pronoun matched the object rather than the subject, regardless of whether the pronoun was null or overt, suggesting a lack of sensitivity to pronoun type. No differences across groups were found in the offline naturalness rating task. The authors argue that the finding that the re-exposed group, who had been in a Spanish-only environment for only a week, did not differ from the Spanish monolingual controls suggests that it is unlikely that any permanent changes had occurred to the native grammars of these speakers. The authors furthermore take the absence of evidence for attrition in the offline task as evidence for the idea that attrition affects the ability to process interface structures, but does not affect knowledge representation. Crucially, however, earlier studies on the same linguistic phenomenon, specifically Tsimpli et al. (2004) on another pro-drop language (Italian), did find attrition in the form of overgeneralization of overt pronouns in contexts where Italian monolinguals would use a null pronoun (i.e., with subject antecedents) using an offline antecedent selection task. Note, however, that it is quite difficult to know whether attrition affects grammatical representation on the basis of comparing performance in online versus offline tasks, as neither allows access to the brains of speakers to measure linguistic competence. As argued by White (2023), essentially all experimental tasks are measures of performance and “both offline and online measures can be used to determine the nature of linguistic representations, as well as processing considerations” (White, 2023, p. 334). We return to this discussion in Section 8 and show how the findings of the current study indicate differences in grammatical representation in the mental grammars of our attrited participants.

## 3 The importance of L1-L2 overlap for attrition

A well-supported finding in attrition research is that L1 forms that have no analogous forms in the L2 are more easily preserved than L1 forms that are in competition with L2 forms (Altenberg, 1991; Köpke, 1999, 2002; Pelc, 2001; Gürel, 2004, 2007; Paradis, 2007; Tsimpli, 2007). We can illustrate the importance of L1-L2 overlap using the interpretation of pronouns in Turkish and English as examined in Gürel (2002, 2004) and Gürel and Yılmaz (2011). Languages differ in the syntactic-semantic constraints on the interpretation of pronouns. The Turkish pronoun *o* functions differently from English pronouns *him/her/they*: while English allows bound interpretations (*he* can refer to *Burak* in (2)), this reading is not possible for the Turkish pronoun *o*.

2. Burak_i_  [o_*i/k_-nun zeki     ol-duğ-u]-nu      düşün-üyor.
  Burak   s/he-gen intelligent be-nom-3sgposs-acc  think-prg
  ‘Burak_i_ thinks that s/he_i/k_ is smart’

In addition to overt pronouns, Turkish also allows null pronouns and the anaphoric pronominal *kendisi* in subject position, while no such counterpart exists for English. To examine the potential effect of English on the interpretation of Turkish *o*, Gürel (2002, 2004) tested Turkish native speakers who were near-native speakers of English and immigrants to North America, as well as native controls in Turkey. The author reports that while the L2 English group and the Turkish monolingual control group did not behave any differently in their interpretation of null pronouns and *kendisi*, the L2 English group chose a bound interpretation of *o* significantly more often than the control group. Gürel (2002, 2004) therefore concludes that competition with an L2 form is needed for attrition to occur. Following the same reasoning, the findings in Tsimpli et al. (2004) also support the idea that structural overlap is needed for attrition to occur. In pro-drop languages like Italian (and Spanish, discussed in Section 2), null pronouns (*pro*) refer to subject antecedents, typically the topic of the sentence, while overt pronouns tend to refer to object antecedents. Following the syntactic analyses in Cardinaletti and Starke (1994) and Grimshaw and Samek-Lodovici (1998), Italian pronouns are argued to be specified for [+Topic shift]. Overt pronouns in non pro-drop languages like English can refer to subjects *and* objects and are not specified for this discourse feature. Because English and Italian both use overt pronouns, L2 English can cause Italian overt pronouns to become optionally underspecified for [+Topic Shift] in the grammars of Italian native speakers who have become dominant speakers of English. In consequence, attriters may over-accept and use overt pronouns in their pro-drop L1, allowing them for both subject and object antecedents. This is exactly what Tsimpli et al. (2004) found: attriters overgeneralized overt pronouns in contexts where Italian monolinguals would use a null pronoun. The interpretation of Italian null pronouns, however, was not affected, as there is no L2 counterpart.

Similarly, Chamorro et al. (2016) tested potential attrition on the use of the object marker *a*, which in Spanish is required to precede a direct object when the object is animate and specific. The results show no signs of attrition in the Spanish of near-native speakers of English in the United Kingdom. Because the participants were the same as in Chamorro et al. (2016) on the interpretation of over pronouns, Chamorro and Sorace (2019) compare the performance in the two studies and attribute the different results to the fact that the distribution of null and overt pronouns involves the external interfaces while the use of the object marker *a* is driven by semantic factors (animacy and specificity) and therefore involves the internal interfaces. However, an alternative explanation for the lack of attrition with the use of *a* is the absence of an L2 analogous form. Crucially, English does not allow differential object marking and therefore L2 properties cannot possibly influence the [+animate] and [+specific] feature of the L1 grammar. English does have overt pronouns but their use differs from pronouns in null-subject languages, causing competition between L1 and L2 forms.

The need for L1-L2 analogous forms as a prerequisite for attrition has also been supported by Iverson (2012). Using online and offline acceptability and interpretation judgment tasks, Iverson (2012) examined the grammar of a native Brazilian Portuguese speaker who by the time of testing almost exclusively spoke his L2 Chilean Spanish. The participant was tested on a range of phenomena at the external interfaces, internal interfaces and phenomena pertaining to narrow syntax. Iverson (2012) found that the main predictor for attrition was not whether the property pertained to the external interfaces, as would be predicted by the IH, but whether Brazilian Portuguese and Chilean Spanish share properties. The author furthermore argues that the speaker’s grammar is qualitatively different from monolingual L1 grammars, as his grammar reflected convergence with the L2 grammar in all linguistic structures where the L1 and the L2 differed.

To conclude, research has focused on whether attrition can cause structural changes to native grammars and if so under which conditions. Although grammatical attrition is likely to be rare, syntactic restructuring has been attested and is more likely to take place as the result of long-term co-activation of a language system that has analogous forms. More research with a broader variety of linguistic structures, language combinations and a combination of various experimental tasks is needed to provide additional insights. The current study tests whether the existence of analogous forms in the L1 and L2 can lead to grammatical restructuring of the L1 using another phenomenon at the syntax-discourse interface, namely the use of Clitic Left Dislocation. We are comparing the L1 Romanian grammars of speakers whose L2 does have an analogous form (Italian) to speakers whose L2 does not use CLLD (English). The properties of CLLD in Romanian and Italian will be discussed in the next section.

## 4 Clitic left dislocation in Romanian and Italian

This paper focuses on two types of A-bar movement of an object into the left-periphery: Contrastive Topic Fronting and Contrastive Focus Fronting, as shown in (3) and (4) respectively.

3. Topic Fronting
  Q: What did you do with the couch and the table?
  A: [The couch]_i_ I put *t*_i_ in the living room, but the table broke during transportation.
4. Focus Fronting
  Q: You put the table in the living room, right?
  A: [THE COUCH]_i_ I put *t*_i_ in the living room, not the table.

In both sentences, the fronted object receives a contrastive interpretation (López, 2009) where “the couch” is contrasted to “the table” mentioned in the previous sentence. Following López, the two constructions in (3) and (4) can be differentiated by the discourse property [± anaphor]. In (3), the object is an example of a discourse anaphor, as the dislocate “the couch” has an antecedent in the immediate discourse (a local antecedent, see Villalba, 2000) and the answer elaborates on the previous sentence by contributing new information about what happened to the couch. In (4), the dislocate “the couch” does not have an antecedent (it is not mentioned in the immediate discourse).^1^ While in English the dislocated object is not doubled by a preverbal clitic in either (3) or (4), in Romanian and in Italian such sentences can be expressed using Clitic Left Dislocation. This section examines the cross-linguistic differences associated with Clitic Left Dislocation (CLLD), an example of which is shown in (5a) for Romanian and in (5b) for Italian.

5. [+specific, +anaphor]
  Q: What did you do with the couch and the table?

- [Canapeaua]_i_  am pus-*(**o**)     în sufragerie,  dar masa    s-a    rupt    în timpul transportului.
  Couch.def   have put-cl.acc.f.sg in living-room but table.def refl-is broken in time   transportation.
  ‘The couch I put in the living room, but the table broke during transportation.’
- [Il divano]_i_ *(**l**’)     ho   messo in soggiorno, ma il  tavolo si   è rotto   durante il  trasporto.
  The couch cl.acc.m.sg   have put   in living-room but the table    refl  is  broken during the transportation
  ‘The couch I put in the living room, but the table broke during transportation.’

In both languages, the dislocated object *the couch* is doubled by a preverbal clitic. However, different conditions underlie the use of clitics with left dislocation in Italian and Romanian, as different features are involved. The two relevant features are *specificity* ([± specific]) and *discourse anaphoricity* ([± anaphor]), the exact mechanisms for which are discussed in Smeets (2022, 2023). In Romanian, only dislocated objects that have a specific referent participate in CLLD [Cornilescu and Dobrovie-Sorin (2008); Avram and Coene (1999); Smeets (2022, 2023)]. In (5), the speaker has a specific couch in mind. However, if we look at clitic use in a scenario where the dislocate is non-specific, as is the case for *a red skirt* in (6) and (8), we see that clitics are not allowed in Romanian. Italian CLLD, on the other hand, is used with both specific and non-specific objects (compare (5b) to (6b)), as Italian CLLD is not constrained by specificity.

6. [−specific, +anaphor]
  Q: Did you find a red skirt and a pair of boots?

- O fustă roșie (*o)      caut   deja     de două luni, dar am     găsit o pereche de ghete negre.
  a skirt   red  cl.acc.f.3sg  search already for two  months but have.1sg found a pair    of boots black
  ‘I’ ve been looking for a red skirt for two months, but I did find a pair of black boots.’
- Una gonna rossa *(**la**)     cerco     già      da   due mesi,    però ho     trovato un paio di stivali neri.
  a   skirt   red  cl.acc.f.sg  search.1sg already  since  two  months but have. 1sg found  a  pair  of boots  black.
  ‘I’ve been looking for a red skirt for two months, but I did find a pair of black boots.’

In Italian, however, CLLD is restricted to discourse topics and cannot be used with contrastive focus fronting, as the ungrammaticality of the clitic shows in (7b). Romanian CLLD, on the other hand, is used with both topic ([+anaphor]) and focus ([−anaphor]) fronting, as shown in (5a) and (7a).

7. [+specific, −anaphor]
  Q: You put the table in the living room, right?

- CANAPEAUA     am pus-*(**o**)        în sufragerie,   nu  masa.    Masa      s-a      rupt  în timpul transportului.
  couch-def     have.1sg put-cl.acc.f.3sg in living-room not table-def table-def   refl-has broken in time  transportation
  ‘The couch I put in the living room, not the table. The table broke during the transportation.’
- Il   DIVANO (*l’)       ho      messo in soggiorno,   non il   tavolo. Il tavolo si    è rotto durante  il   trasporto.
  The couch     cl.acc.m.sg    have.1sg put    in living-room not the table.  the table    refl  is broken during the transportation.
  ‘The couch I put in the living room, not the table. The table broke during the transportation.’

In the context in (8) neither Italian nor Romanian uses a clitic. A clitic is not allowed in Italian because the fronted object is [−anaphor] and it is not allowed in Romanian because the dislocate is [−specific].

8. [−specific, −anaphor]
  Q: Weren’t you looking for a red sweater? I saw some nice ones at H&M.

- O FUSTĂ roșie o        caut,        nu o cămașă roșie.
  A SKIRT   red     cl.acc.f.3sg seek-for.1sg not a sweater red.
  ‘I am looking for a red skirt, not a red sweater.’
- Una GONNA rossa (*la)       cerco,       non una maglietta rossa.
  A     skirt     red   cl.acc.f.3sg look-for.1sg, not a    sweater   red.
  ‘I am looking for a red skirt, not a red sweater.’       Smeets (2023), examples 4–7.

Although specificity is typically assumed to be a semantic feature on noun phrases, specificity is not marked on determiners or nouns in Romanian, Italian and English. In Romanian the distribution of clitics is dependent on whether the fronted object is [± specific] and whether an (indefinite) noun is interpreted as specific or non-specific depends on whether there is a specific referent available in the discourse context. Similarly, whether or not a constituent is discourse anaphoric requires the reader or listener to keep information from the previous discourse in working memory. Therefore, in both Romanian and Italian, the presence or absence of a clitic depends on changing contextual information. The distribution of clitics in Romanian and Italian is summarized in Table 1.

**Table 1:** Distribution of resumptive clitics in Italian and Romanian.

		[+ anaphor]		[− anaphor]		Property
		[+ specific]	[− specific]	[+ specific]	[− specific]	
1	Italian	✓	✓	χ	χ	anaphoricity
2	Romanian	✓	χ	✓	χ	specificity

## 5 The attrition via acquisition model: predictions for CLLD

The current study examines the use of CLLD by Romanian first generation immigrants to either Italy or an anglophone country. The hypotheses and results will be interpreted in light of the Attrition via Acquisition (AvA) model (Hicks and Domínguez, 2020), a formal model of grammatical attrition which provides a testable hypothesis for the conditions where L2 properties may change mature L1 grammars.

The model is developed within the generative framework, which assumes that differences between languages are expressed in the features they select from an innately available universal set of features and the way they apply those features to lexical items and morphemes (as in the Minimalist Program, Chomsky, 2000, 2001 et seq.). The AvA addresses the question of whether formal features that are set in early childhood can be reset due to influence of L2 features in speakers with reduced access to and use of their L1. In order to explain changes to feature representations, Hicks and Domínguez (2020) elaborate on the Feature Reassembly Hypothesis (FRH, Lardiere, 2008), a prominent theory in generative language acquisition research which examines the role of L1 transfer into L2 grammars to explain relative difficulty and success in L2 acquisition. Specifically, the FRH is developed around the fine-grained differences across languages on how they encode grammatical features. The FRH predicts that L2ers at the initial stage transfer the features associated with specific lexical items into the L2 grammar. The task of an L2er then involves adjustments to features on morphemes or lexical items that were incorrectly transferred from the L1 grammar. Applying the FRH to attrition, the Attrition via Acquisition (AvA) model argues that grammatical attrition consists of adjustments to L1 features on individual morphemes that are transferred from the L2 in situations of L1-L2 overlap. The AvA therefore predicts L1 grammatical attrition to be possible when there are analogous forms in the L1 and L2 that yet differ to some extent, in line with the findings discussed in Section 3.

Crucially, however, while the presence of L1 features in L2 grammars is extremely common and well-attested, the presence of L2 features in L1 grammars is certainly much rarer. To explain the rarity of grammatical attrition, Hicks and Domínguez follow Lidz and Gagliardi (2015)’s theory on L1 acquisition to discuss how grammatical properties of a grammar that has reached maturity can become less stable and open for the intake of new grammatical properties. The AvA model assumes a unified mechanism for acquisition and attrition where L1 attrition engages in the same acquisitional mechanisms as L1 and L2 acquisition. The theory decouples linguistic input from acquisitional intake, which is the information the mind actually extracts from the input. Hicks and Domínguez propose that when extensive L2 input is accompanied with a reduction in L1 input, the so-called ‘inference engine’ may be reopened to take in new features that update the existing L1 grammar. L2 interference, a prerequisite for the eventual alternation of L1 representations, can only occur for linguistic phenomena where there is overlap between the L1 and the L2 but where the L2 assumes different values (features) for corresponding linguistic items. Specifically, Hicks and Domínguez (2020, p. 156) predict grammatical attrition to be possible under the following circumstances:

– The L2 is close, yet not identical, to the speaker’s first language. Specifically, the L1 and the L2 allow a certain syntactic construction but use them in different situations. Hence, prolonged exposure to the L2 can alter L1 feature-form mappings, but only if the L2 allows for the same syntactic construction as the L1.
– The changes in the L1 grammar do not involve a loss of options from the L1 grammar, or replacement of L1 features by L2 features. Instead, options from the L2 grammar supplement the existing grammar, leading to L1 restructuring.

As pointed out by Schmid and Köpke (2017b), a feature reassembly approach to L1 loss where L2 features influence the feature bundles of the L1 has previously only been applied to contexts of heritage language acquisition, where developing L1 grammars whose feature representations are shown to be weaker or incomplete are affected by L2 features (Putnam and Sánchez, 2013) and to contexts of contact-induced change involving two minimally different varieties of the same language (Cuban and Peninsular Spanish, see Domínguez and Hicks, 2016). While the AvA predicts that grammatical attrition is favored when the L1 and L2 are typologically more similar, it also predicts that similar (but not identical) comparative behavior in the L1 and L2 provides a sufficient condition for attrition to occur. Hicks and Domínguez (2020) are the first to apply the Feature Reassembly approach to attrition in late L2 learners of a different (mutually unintelligible) language.

Specifically, the authors illustrate the applicability of the AvA model with data from previously reported findings on the realization and interpretation of pronominals in adult first generation immigrants. As discussed in Section 3, overt pronouns in pro-drop languages like Italian and Spanish have been argued to have a [+Topic shift] feature, where the use of an overt pronouns indicates an interpretation away from the discourse topic (typically the subject). Overt pronouns in non pro-drop languages like English do not have a discourse feature, as pronouns are used with both topic and non-topic antecedents. The existence of overt pronouns in both languages, albeit used in different contexts, meets the requirement of the AvA for grammatical attrition to be possible. The properties of the overt pronoun of the L2 can be transferred onto the L1 and affect the use and interpretation of pronominals in attrited native speakers of a pro-drop language. The presence of overlap in the use of overt subject pronominals in both English and Italian/Spanish can cause attriters to associate the feature specifications of English pronominals (which lacks a [+Topic Shift] feature) with the corresponding pronominal of Italian/Spanish. In consequence, overt pronouns are also used in contexts where there is no topic shift. The authors argue that “continued processing of L2 input that invokes both UG and the L1 in updating the advanced L2 grammar allows for the possibility that acquired morphosyntactic features of the relevant L2 lexical item ‘update’ the L1 grammar” (Hicks and Domínguez, 2020, p. 157).

To examine the validity of the AvA and to further improve the model, it is important to apply the predictions to new linguistic contexts and language combinations. To date, the application of the AvA has mostly focused on studies examining the interpretation of overt pronouns in L1 pro-drop languages [but see recent work by Baker (2024) who examined a variety of morpho-syntactic phenomena, finding different types of changes to L1 grammars for some speakers and for some linguistic phenomena]. It is relevant to note that for the use of overt pronouns in pro-drop languages, it is hard to convincingly conclude that the attested attrition effects are due to L2 transfer in the absence of another group whose L1 and L2 work the same. As pointed out by Montrul (2008), the over-acceptance of overt pronouns with topic antecedents can also be due to the alleged complexity associated with the syntax-discourse interface, as predicted by the IH. Furthermore, attrition effects independent of L2 transfer have also been attested in the form of simplification, where marked forms are replaced by unmarked forms (Schmid, 2002, p. 13). Applying this reasoning to the use of subject pronouns, the overuse of overt pronouns in contexts without topic shift could also be the result of those speakers resorting to unmarked values by removing the [+topic shift] feature from overt pronouns. For this reason, it is important to also include an experimental group where L2 transfer cannot occur, which may be impossible for the interpretation of overt pronouns, assuming overt pronouns exist in all languages. The cross-linguistic differences between Romanian and Italian and the lack of CLLD in English form an ideal test case to disentangle L2 transfer effects as predicted by the AvA model from other factors potentially causing changes to attrited grammars.

## 6 Current study

Our discussion so far has focused on the effects of L2-driven factors on attrition and the importance of analogous forms in the L1 and the L2 for attrition to occur. To further test the AvA, we compare the use of CLLD by Romanian native speakers who are living in an English-dominant environment (no L2 transfer possible) to Romanians living in Italy (L2 transfer possible), as attrition is predicted to be possible only for the latter group. Specifically, L2 options are predicted to supplement the L1 grammar. This means that grammatical attrition does not involve a complete loss of L1 forms but a fluctuation between the grammatical options from the L1 and the L2.^2^ For the use of CLLD, attriters who are L2 speakers of Italian are expected to supplement their L1 Romanian grammar with options available in Italian and therefore allow clitics when the fronted object is a non-specific topic. Although the AvA would not predict any changes to the mental representation of Romanians in anglophone countries, data from the L2 English group can provide insights into whether there are causes to attrition that are independent from L2 transfer, for example due to reduced activation of the L1 grammar possibly which may cause inconsistent or inefficient processing. The predictions following the AvA are summarized in Table 2.

**Table 2:** Expected use of CLLD per condition according to the predictions of the AvA.

		[+ anaphor]		[− anaphor]	
		[+ specific]	[− specific]	[+ specific]	[− specific]
1	Romanians in Italy	✓	✓	✓	χ
2	Romanians in anglophone countries	✓	χ	✓	χ

Since we are interested in examining whether attrition affects knowledge representation, we tried to reduce processing difficulties as much as possible by using two untimed tasks, an Acceptability Judgment and a Written Elicitation task. We expect Romanians in Italy to accept clitics with non-specific topics in the Acceptability Judgment task and to use clitics in this context in the Written Elicitation task. We furthermore expect them to continue to use and accept clitics with specific foci.

### 6.1 Methods

#### 6.1.1 Participants

A total of 95 participants completed the experimental tasks, either in Italian or in Romanian (see participant details and background information in Table 3). Participants were recruited by research assistants who were in-group members of Romanian immigrant communities in Italy or the US and Canada. Prior to participation it was ensured that none of the participants spoke another language with CLLD (e.g., Spanish, Greek or Bulgarian). In addition to the Acceptability Judgment task and the Written Elicitation task, which were always presented in this order, participants filled out an extensive background questionnaire adapted from Schmid and Dusseldorp (2010) to gain more information on extralinguistic factors that may affect attrition rate. Note that the questionnaire included a question asking about participants’ current age, the age at which they started to acquire the L2 and their length of residence in the country where the L2 is spoken. Since English is a language that is widely taught in elementary schools as a second language, the age of onset of the L2 English group was much lower than that of the L2 Italian group. However, since any signs of L1 attrition likely only start to occur when immersed in an environment where the L2 is spoken, we used the age of arrival instead, by subtracting the length of residence from their current age. The questionnaire furthermore consisted of several questions related to language use as well as those related to the participant’s language and cultural attitudes. Following the suggestions in Schmid (2011, p. 214), it is desirable to reduce the number of responses from each individual question by taking an average over various questions that can be grouped together. Note, however, that there are no specific guidelines available for coding questions into such broader categories. I decided to group together various questions that relate to relative language proficiency, questions that ask about external language use both inside and outside the home and a third group of questions that relate to internal language use, which is argued to be indicative of someone’s linguistic and cultural affiliation (Schmid, 2011). Specifically, *Relative language proficiency* indicates an average score participants gave to nine questions, listed below, related to self-reported relative language proficiency. Participants were asked to indicate whether they were more proficient in the L1 (Romanian) or the L2 (Italian/English) using a 1–5 scale where 1 indicates “only Italian/ English,” 2 “mostly Italian/English,” 3 “both,” 4 “mostly Romanian” and 5 “only Romanian.” An average score of 3.0 indicates that speakers had equal proficiency in both languages.

1. In which language do you have the largest vocabulary?
2. In which language do you have no pronunciation issues?
3. Which language are you able to use/understand intuitively?
4. In which language are you familiar with various dialects/slang?
5. In which language do you have an in intuitive feeling of what is “correct”/ “incorrect”?
6. Into which language are you able to translate?
7. In which language can you understand and make jokes?
8. In what language do others consider you a native speaker?
9. In which language can you express yourself more easily?

**Table 3:** Background information, showing mean (range) for age, age of arrival, length of residence, relative language proficiency and language use.

	Italian monolinguals	Romanian monolinguals	L2 English	L2 Italian
Number of participants	18	17	23	37
Age	33.4 (24–53)	33.4 (24–51)	51 (22–69)	39.44 (23–58)
Age of arrival			29.7 (18–47)	23.03 (11–40)
Length of residence			21.3 (4–21)	16.4 (3–20)
Relative language proficiency			3.65	3.53
External language use			3.16	2.82
Internal language use			3.43	3.38

*External language use* shows the average response to seven questions that asked participants what language(s) they speak with (1) friends, (2) daily basis, (3) partner, (4) pets, (5) work, (6) relatives, and (7) at the store. The score for *Internal language use* consists of six questions asking participants in which language they (1) think, (2) dream, (3) count, (4) swear, (5) use when emotional and (6) use when upset. These questions used the same five-point rating Likert scale. The average response to each group of questions and each L2 group is shown in Table 3.

As can be observed in Table 3, the length of residence in the L2 country is somewhat longer for the L2 English group, although speakers in both groups, on average, have been living in the country where the L2 is spoken for a substantial number of years. Note that the L2 Italian group included speakers who immigrated during early adolescence, while the earliest age of arrival for the L2 English group is 18. We will get back to effects of onset of immigration in Section 7. The two groups are highly comparable in their responses to the sociolinguistic variables, with both groups stating to only be slightly more proficient in their L1 than their L2 and to use their native language slightly more internally (which has been argued to reflect their cultural and linguistic identity).

#### 6.1.2 Experimental tasks

In the Acceptability Judgment task, participants were asked to judge the acceptability of sentences with left dislocated word orders on a 6-point rating scale where 1 indicated highly unacceptable and 6 highly acceptable in the context in which they were presented. Experimental trials were like the examples in (5) to (8) in which the contexts, questions and answers were presented in Italian for the Italian monolingual control group and in Romanian for the Romanian monolinguals and the two attriter groups. All experimental trials can be found.^3^ The experiment consisted of 64 target items which differed by three factors: *Discourse* and *Clitic*, which are within-item factors, and *Specificity*, which is a between-item factor. The factor *Discourse* had two levels: in the Topic context, as in (5) and (6), the left dislocated object was discourse anaphoric and in the Focus context, as in (7) and (8), the fronted object was not discourse anaphoric. For Specificity, the fronted object was either specific, as in (5) and (7), or non-specific, as in (6) and (8). For Clitic, there was either a clitic or no clitic.

**Figure 1:**
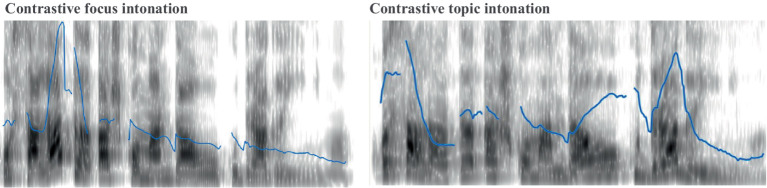
Intonations used for answers in contrastive focus (left) and contrastive topic (right) contexts. Example shown for the sentence “O salată aș comanda cu dragă inimă” (a salad I would like to order).

Stimuli were presented both in written and auditory form to ensure that participants processed the sentences with the intended intonation. To ensure the pronunciation of the question-and-answer pairs was most natural given the context provided, each full experimental trial, including context, question and answer, was recorded by both a female and a male native speaker of Romanian. To create the experimental trials, recordings of the contexts and the answers were then taken from the female voice and the questions were taken from the male voice (in alignment with the gender of the characters in the story). The female was also a linguist who helped ensure that the pronunciation used was most natural given the intended interpretation. As illustrated with an example in Figure 1, fronted constituents in the Focus contexts received a high tone (H*) followed by a default low tone [following Jitcă et al. (2015) who identified this intonational pattern for Romanian contrastively focused constituents]. Contrastive topic configurations were associated with a ‘rise-fall-rise’ intonation, as argued by Büring (2002) for English and in agreement with judgments from native Romanian research assistants for Romanian.

**Figure 2:**
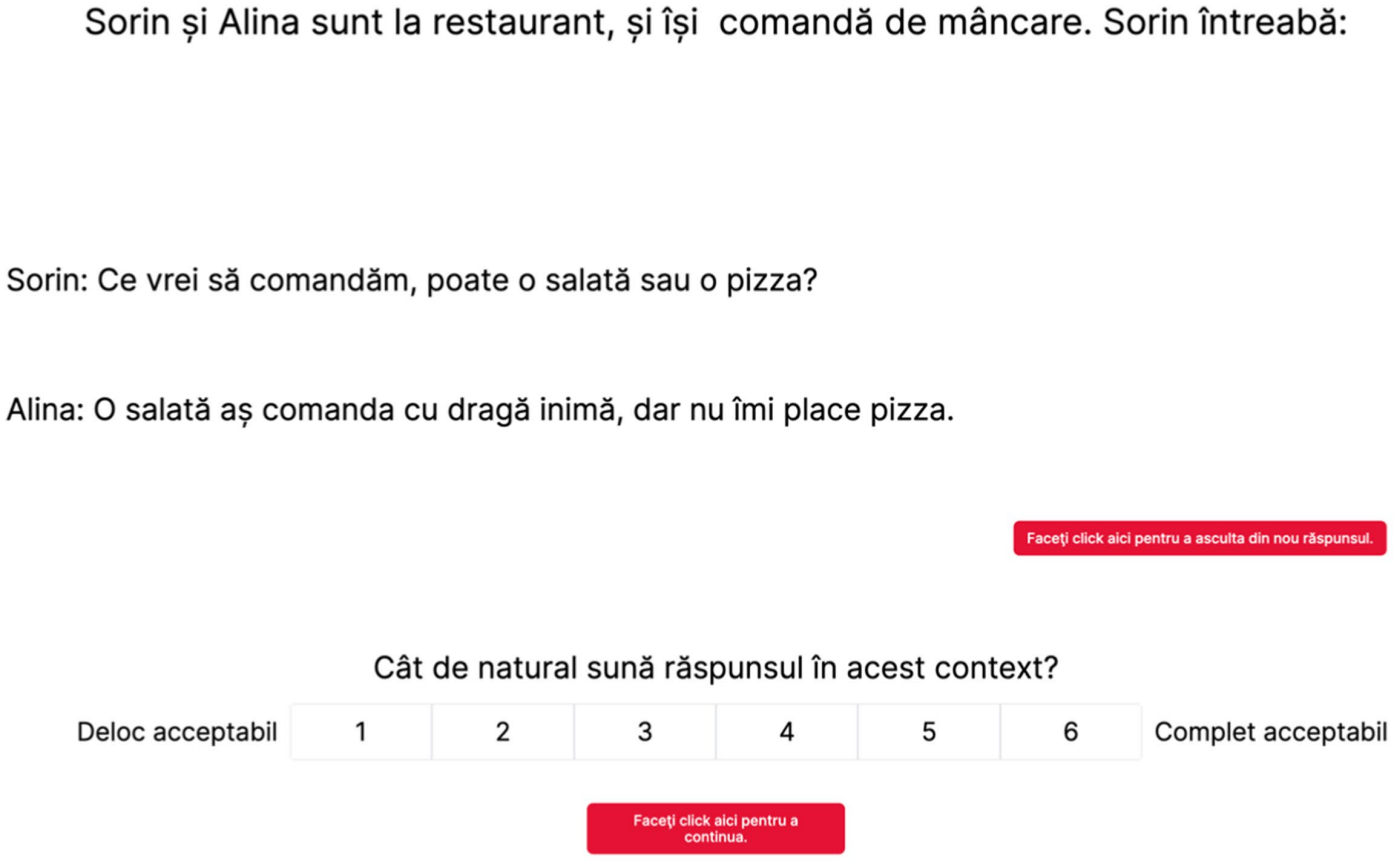
Example trial from Gorilla experiment builder.

Each experimental trial was presented as follows: the context automatically appeared, after which the question-and-answer pair was shown, both in written and spoken form. Thereafter, the acceptability judgment scale appeared asking participants to rate on a scale of 1 to 6 how natural the answer sounded in the context provided. An example is shown in Figure 2.

In the Written Elicitation task (WET), participants were asked to complete sentences that were partially left blank and were instructed to complete the sentence using a part of a word or alternatively one, two or three words. The parts left blank aimed at eliciting a verb alone or a clitic and a verb. The WET consisted of 16 target items, four of each in the four possible conditions as illustrated in (9)–(12), varying the factors [± specific] and [± anaphor]. For each example in (9)–(12), the first answer illustrates a target sentence for Romanian and the second for Italian. In the actual experiment, the context, question and answer were of course completely shown in either Romanian or Italian. The experimental items were randomly interspersed with 53 filler items, where participants were asked to complete the sentences using tense, number and person inflections, prepositions and determiners. Each item started with a short context followed by a question-answer pair.

9. [+ specific, +anaphor]
  Livio is looking for someone who can take his granny’s cat and dog as she cannot take care of them anymore. Livio asks Silvia:
  Q: Would you maybe want to adopt the cat or the dog?
  A: Pisica *o voi adopta/ as adopta-o/ o pot adopta* cu drag,     dar nu avem loc pentru un câine.
  A: Il gatto *lo adotterei/lo prenderei*     volentieri, ma non abbiamo spazio per un cane.
  The cat *(CL) would/will/can.1sg adopt/take* happily, but I do not have space for a dog.
10. [+ specific, −anaphor]
  Anna and Beatrice are talking about Lea and Gianni who recently got married. Anna says to Beatrice:
  Q: They have visited the Virgin Islands if I remember correctly.
  A: Insulele MALDIVE _____*le-au*___vizitat în luna de miere, nu Insulele Virgine.
  A: Le MALDIVE *hanno* visitato per il viaggio di nozze, non le isole Vergini.
  The Maldives *(CL) have.3pl* visited for the honeymoon, not the Virgin Islands.
11. [−specific, +anaphor]
  Alessandra is in the library but she is not sure what she wants to read and she goes to the librarian to ask for recommendations. The librarian says:
  Q:  Would you like to read a book about airplanes or one about cars?
  A: O carte despre avioane ____*as citi*______     cu plăcere, dar mașinile nu mă interesează.
  A: Un libro sugli aeroplani *lo leggerei*    con piacere, ma le macchine non mi interessano.
  A book about airplanes *(CL) will/would.1sg read* with pleasure, but I am not interested in cars.
12. [−specific, −anaphor]
  Elena will go shopping this weekend because she has a date. Her friend tries to be helpful and says:
  Q: Weren’t you looking for a black shirt? I saw some cute ones at Zara.
  A: O ROCHIE neagră _______*caut*_______, nu o    cămașă   neagră.
  A: Un VESTITO nero ______ *cerco*______, non una maglietta nera.
  A black dress *(CL) search.1sg.*, not a black shirt.

### 6.2 Results

#### 6.2.1 Acceptability judgment task

The results were plotted for each group separately to examine systematic patterns within the (interlanguage) grammars themselves (following Bley-Vroman, 1983). All felicity ratings were analyzed using cumulative link mixed effects models (Christensen, 2014). The models include fixed effects for the categorical predictors *Clitic, Specificity, Discourse* and their interactions and random effects for Participant and Item. Each contrast was centered using sum coding. Random-effect slopes were based on a maximal model that allowed convergence, following Barr et al. (2013).

**Figure 3:**
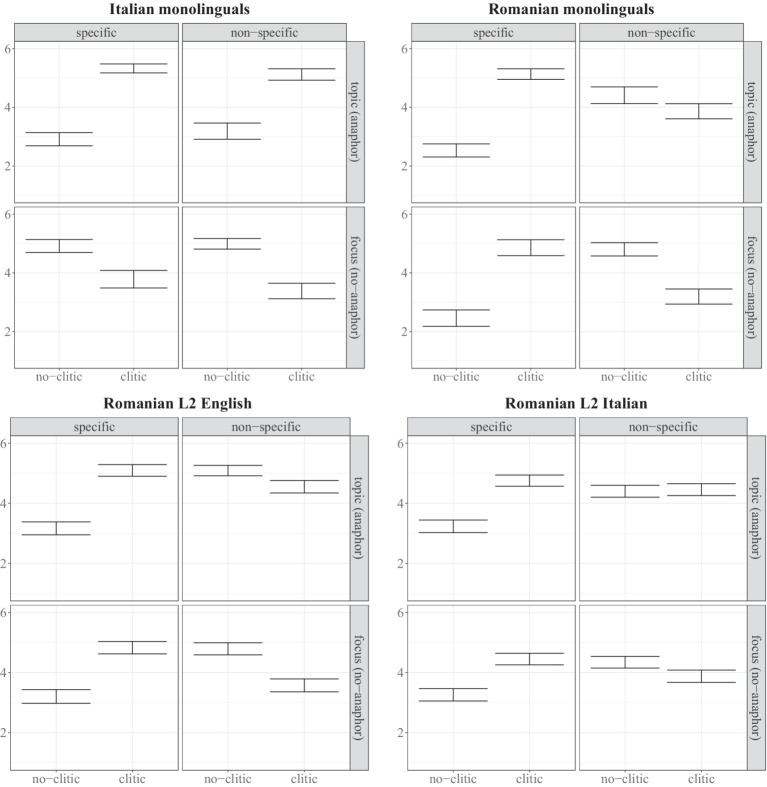
Acceptability judgments from Italian monolinguals, Romanian monolinguals, L2 English and L2 Italian speakers with means and error bars showing 95% confidence intervals.

Figure 3 shows the results from the Italian and Romanian monolinguals, Romanians in anglophone countries, and Romanians in Italy, and Table 4 the outcomes of the cumulative link mixed effect models for each of the four groups. Each line in the table starts with the label of the predictor in bold (e.g., Clitic) followed by the levels that were contrasted. For example Clitic No-Yes shows the effect of the absence vs. presence of the clitic, where the first level shows the baseline.

**Table 4:** Acceptability judgments from Romanian monolinguals, L2 English and L2 Italian speakers.

Effects on acceptability judgment				
	Italian mon	Romanian mon	L2 English	L2 Italian
Predictor	Estimate(SE), *p*-value	Estimate(SE), *p-*value	Estimate(SE), *p-*value	Estimate(SE), *p-*value
Clitic No-Yes	**0.67 (0.24), *p* = 0.006**	**1.33 (0.41), *p* = 0.001**	**3.20 (0.46), *p* < 0.001**	**2.27 (0.45), *p* < 0.001**
Specific Spec-NonSpec	−0.10 (0.24)	0.89 (0.50)	**2.92 (0.54), *p* < 0.001**	**1.61 (0.40), *p* < 0.001**
Anaphor Top-Foc	−0.04 (0.19)	−0.49 (0.28)	0.01 (0.18)	0.05 (0.18)
Clitic No-Yes: Specific Spec-NonSpec	**−0.92 (0.47), *p* = 0.048**	**−7.48 (1.36), *p* < 0.001**	**−4.15 (0.65), *p* < 0.001**	**−2.19 (0.45), *p* < 0.001**
Clitic No-Yes: Anaphor Top-Foc	**−6.63 (1.16), *p* < 0.001**	**−1.29 (0.51), *p* = 0.012**	−0.55 (0.30)	**−0.53 (0.27), *p* = 0.05**
Specific Spec-NonSpec: Anaphor Top-Foc	−0.48 (0.36)	−0.23 (0.90)	−0.46 (0.32)	−0.09 (0.26)
Clitic No-Yes: Specific Spec-NonSpec: AnaphorT-F	−0.00 (0.56)	−0.03 (0.11)	−0.47 (0.47)	−0.25 (0.38)

Each column shows the estimate for each predictor with the standard error in parentheses. Significant effects are shown in bold, and *p*-values are provided.

Discourse has the strongest effect in Italian, as shown by the interaction between Clitic and Discourse (Topic vs. Focus), and Specificity has the strongest effect in Romanian monolinguals, as shown by the interaction between Clitic and Specificity [see Smeets (2023)] for more details on the monolingual results, including a model that directly compares the two groups showing they are significantly different. The same significant interaction between Clitic and Specificity is found for the L2 English and L2 Italian groups, who rate clitic sentences as more acceptable with specific objects and no-clitic sentences as more acceptable with non-specific objects. Furthermore, there is a marginally significant interaction between Clitic and Discourse for the L2 Italian group, suggesting an effect of Discourse on the ratings of clitic vs. no-clitic sentences. As shown in Figure 3, L2 Italian speakers as a group rate the acceptability of clitic and no-clitic sentences in Romanian as equally acceptable with fronted non-specific topics.

To summarize, Romanian monolinguals as well as L2 English and L2 Italian speakers rate clitic sentences as more acceptable than no-clitic sentences when the fronted object is specific. Additionally, Romanians in Italy rate clitic and no-clitic sentences as equally acceptable in the non-specific topic condition while the other native Romanian groups rate no-clitic sentences as more acceptable in this context. This result is suggestive of L2 influence as clitics with non-specific topics have become acceptable in the L1 of Romanians who acquired Italian. In Section 7 we elaborate on the L2 Italian findings by looking at differences across speakers in this group and show that the equal rating of clitic and no-clitic sentences with non-specific objects is driven by some speakers rating no-clitic sentences as more acceptable (in line the Romanian grammar) and others rating clitic sentences as more acceptable (in line with the Italian grammar).

#### 6.2.2 Written elicitation task

All answers in the Written Elicitation task were manually coded by a native Romanian linguist, who assigned the value “1” to answers with a clitic and “0” when the clitic was absent. For a small proportion of trials participants gave an answer that did not provide a context where a clitic could have been used, such as the word of affirmation *da* or use of verbs where the intended object functioned as an experiencer subject, such as with the verb *place* ‘like’ or verbs in the passive voice. For this reason, 17 items (4.7%) for the Italian monolinguals, 14 items (4.1%) for the Romanian monolinguals and 46 items (4.9%) for the attriter groups were excluded from the analysis.

**Figure 4:**
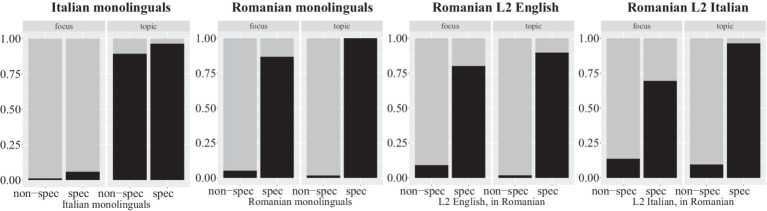
Proportion of clitic use by Italian monolinguals, Romanian monolinguals, Romanian L2 speakers of English and Romanian L2 speakers of Italian.

Figure 4 shows the proportion of clitics used by Italian monolinguals, Romanian monolinguals, Romanian immigrants to anglophone countries and Romanian immigrants to Italy, and Table 5 shows the results of binary logistic regression models. Random effects for Participant and Item were included where possible, aiming for a maximal model that allowed convergence (Barr et al., 2013).

**Table 5:** Written Elicitation task (WET) results from Romanian monolinguals, Romanians in English speaking countries and Romanians in Italy.

	Effects of specificity and discourse on use of clitic			
	Italian monolings	Romanian monolings	L2 English	L2 Italian
Predictor	Estimate(SE), *p-*value	Estimate(SE), *p-*value	Estimate(SE), *p-*value	Estimate(SE), *p-*value
Intercept	0.65 (0.66)	−0.27 (3.43)	0.63 (0.98)	0.60 (0.93)
Anaphoricity Top-Foc	**9.08 (1.95), *p* < 0.001**	9.11 (19.07)	−0.53(6.82)	0.66 (1.42)
Specificity Spec-NonSpec	**2.25 (1.05), *p* = 0.033**	**48.35 (12.03), *p* < 0.001**	**7.4 (1.94), *p < 0*.001**	**9.39 (1.95), *p* < 0.001**
Anaphoricity Top-Foc: Specificity Spec-NonSpec	−0.18 (1.91)	−2.88 (18.75)	−5.53 (3.55)	**−12.65 (4.03), *p* < 0.01**

Showing effects of specificity and discourse on use of clitic: estimate for each predictor with SE in parentheses. Significant effects are shown in bold, and *p*-values are provided.

As can be observed in Figure 4, Italian monolinguals used clitics in both topic conditions, regardless of specificity. Speakers in all three native Romanian groups rarely used a clitic when the fronted object was non-specific. Results from logistic regression models confirm that the odds of using a clitic are significantly greater in the specific conditions than in the no-clitic conditions. Additionally, the L2 Italian group shows a significant interaction between specificity and discourse. In addition to clitics being used more when the fronted object is specific, clitics were also used more in the specific topic condition than the specific focus condition.

#### 6.2.3 Interim conclusion

The results from both the Acceptability Judgment and the Written Elicitation task show no significant effect of attrition for the Romanian native speakers who immigrated to an anglophone country. These participants fully retained the specificity feature associated with CLLD in Romanian. Romanians residing in Italy, however, also accepted and used clitics when the fronted object was [+specific], but discourse anaphoricity, the property that is associated with CLLD in Italian, seems to interfere: in the Acceptability Judgment task the group result showed no difference between clitic and no-clitic sentences with fronted non-specific topics, and in the Written Elicitation task, clitics were not used consistently with specific foci. The differences in performance patterns between the L2 English and the L2 Italian group are in line with the prediction that attrition occurs when individuals are exposed to a syntactic structure that exists in both the L1 and L2 but are nonetheless distinct, as these L2 features are susceptible to competition. Since attrition is typically characterized by individual variation and to further examine whether the group results discussed in Section 6 are driven by specific individuals, we examine effects of language dominance and age of onset of L2 acquisition (using the criteria from Table 3) in Section 7. This section focuses only on the data from the L2 Italian speakers, as L1-L2 differences were only found in this group.

## 7 Effects of age of arrival and language dominance

Attrition is typically characterized by individual variation and the extent at which attrition occurs has been argued to be modulated by (a combination of) various factors, including age of immigration and relative language use.

The most prominent non-linguistic factor for attrition is age of onset of bilingualism: attrition has been shown to be quite common for speakers who immigrate before the onset of puberty (roughly between the ages of 8 and 13 years; Köpke and Schmid, 2004; Pallier, 2007; Bylund, 2009), in particular for morpho-syntactic phenomena (e.g., Montrul, 2008; Montrul et al., 2014). The grammars of post-puberty bilinguals are much more stable and restructuring is argued to be fairly rare (Köpke and Schmid, 2004). Crucially, however, very few studies have looked at the grammars of adolescents, as most research has focused on the grammars of speakers who became bilingual in childhood (heritage language acquisition) or in post-puberty bilinguals (attrition studies; Schmid and Köpke, 2017a). Since the age of arrival (AoA) of the L2 Italian group ranged from age 11 to 40, we have the data to examine potential effects of AoA and to examine whether attrition is more likely in those who immigrated during adolescence.

**Figure 5:**
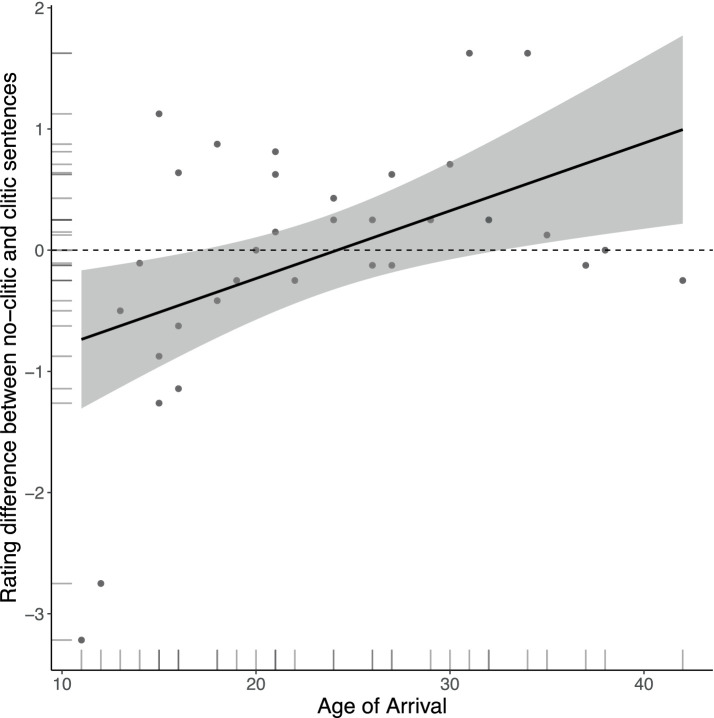
Differences in judgments between no-clitic and clitic sentences in the non-specific topic condition, showing results from Romanian native speakers residing in Italy.

The results of the Acceptability Judgment task showed that Romanians in Italy differed from the other native Romanian groups in how they distinguished between clitic and no-clitic sentences in the non-specific topic condition. This is also the discourse context for which the AvA predicts seeing effects of attrition, as the use of clitics in Italian in this condition could be transferred to Romanian. Figure 3 showed that Romanians in Italy rated clitic and no-clitic sentences as equally acceptable with non-specific topics, while Italian monolinguals preferred clitic sentences and the other Romanian native groups preferred no-clitic sentences in this condition. It is, however, possible that this group effect is driven by some speakers rating no-clitic sentences and others rating clitic sentences as more acceptable. To visualize individual differences and to examine trends in the data on how age of arrival may have affected the ratings of clitic versus no-clitic sentences in the non-specific topic condition, we calculated a new composite dependent variable showing how each individual distinguished between clitic and no-clitic sentences in this condition.^4^ We subtracted each participant’s average rating for sentences without clitics from those with clitics. A negative value therefore indicates a higher rating for clitic sentences and a positive value a higher rating for no-clitic sentences. Figure 5 shows how the difference scores change as a function of age of arrival. The increasing line in the scatterplot, whereby earlier arrivals tend to rate clitic sentences as more acceptable and later arrivals tend to rate no-clitic as more acceptable, suggests that AoA affects acceptability judgments of clitic and no-clitic sentences with fronted non-specific topics. We furthermore observe that more speakers with an earlier AoA, especially those who migrated to Italy before their mid-twenties, had a difference score below 0 (below the dashed line), meaning that clitic sentences were preferred for these speakers.

**Figure 6:**
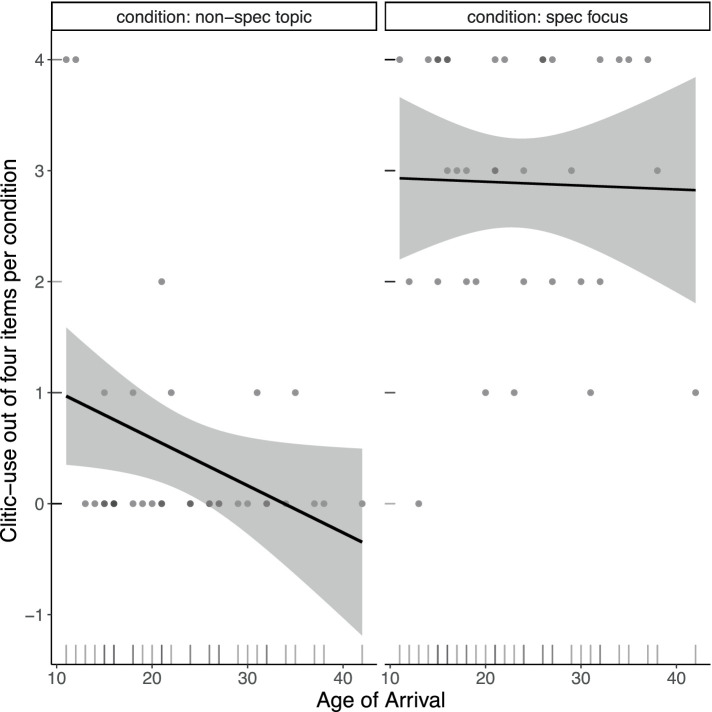
Clitics used by individual participants in the non-specific topic and specific focus conditions, showing results from Romanian native speakers residing in Italy.

Figure 6 shows individual differences in the use of clitics in the Written Elicitation task, plotted against age of arrival. We are showing the results of two conditions where Romanian and Italian differ and where more variability is expected, namely the non-specific topic and specific focus conditions. For sentences with non-specific topics, a high use of clitics suggests attrition, while for sentences with specific foci, a low use of clitics suggests attrition. The results from the non-specific topic condition show that most participants never used a clitic in this condition. However, note that there are two early arrivals (their AoA = 11 and 12) who consistently used clitics in this condition. Their results are likely driving the AoA effect suggested by the trend line in the scatterplot. For the specific focus condition, the relatively flat line suggests that there is no AoA effect for the specific focus condition. In fact, there is much more variability across individuals in their use of clitics in this condition.

**Figure 7:**
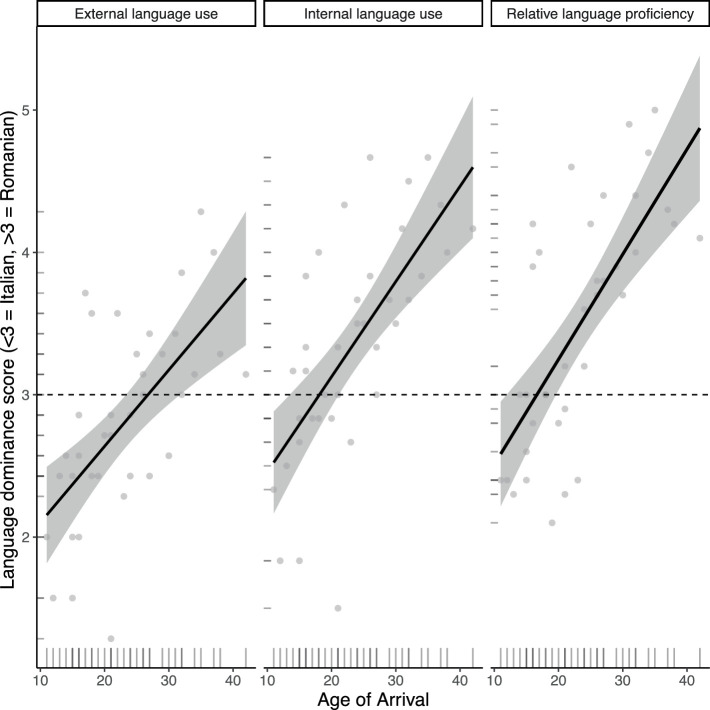
Correlation between age of arrival and language dominance scores for Romanians residing in Italy.

It is important to point out that the AoA effects we observed for the non-specific topic condition can also be due to a difference in language dominance between earlier and later migrants. In fact, the literature on attrition reports a relationship between attrition and language dominance and “… even if a reversal in language dominance is not necessarily followed by attrition, it is most likely that attrition is preceded by such a reversal…” (Köpke and Schmid, 2004, p. 12). Although language dominance is often defined as the relative proficiency in each of a bilingual’s languages, it has been proposed for adult bilinguals that language dominance may be independent from language proficiency (Gertken et al., 2014) and also that dominance is a complex interaction between proficiency and input components (e.g., Montrul, 2015). Because of this and due to the lack of a standardized measure to determine language dominance using the questionnaire we adopted, we define language dominance as a mix between proficiency and exposure/language use criteria. As discussed in Section 6.1, our background questionnaire contained questions on language proficiency, external language use and internal language use. Figure 7 shows the individual average scores for questions on each of these three factors and its correlation with AoA. Based on the five-point scale used in our background questionnaire, an average score lower than 3 indicates a higher proficiency and use of Italian, while a score higher than 3 indicates a higher proficiency and use of Romanian. The plot shows that those individuals who arrived in Italy before their mid-twenties tended to use Italian more than Romanian (“External language use”), while the opposite holds for those who immigrated post-adolescence. Similar AoA effects are observed for “Internal language use” and “Relative language proficiency,” where those who immigrated at a later age use Romanian more than Italian and judge themselves as more proficient in their L1. Note furthermore that for “External language use” about half of our participants use Italian more while the other half uses Romanian more. However, for “Internal language use” and “Relative language proficiency,” more participants showed an average rating above 3 (above the dashed line), suggesting that their linguistic and cultural affiliation is more strongly connected with Romanian. In Section 8.1 we will elaborate on why many of our participants may be more connected to their Romanian identity and claim to be more proficient in Romanian than in Italian despite having lived in Italy for 10+ years.

To summarize, a closer look into the performance of the L2 Italian group revealed that the group results in our experimental tasks were mainly driven by speakers who immigrated to Italy during adolescence and in their early twenties and who were more dominant speakers of Italian. Recall that the AvA predicts grammatical attrition to occur in the form of L2 options being added to the existing L1 options, predicting an increased use of clitics with non-specific topics, as this is where clitics are used in Italian but not in Romanian. For the AJ task we found that earlier arrivals tend to rate clitic sentences as more acceptable than no-clitic sentences (like Italian monolinguals), while the opposite tends to hold for later arrivals. In the WET, however, use of clitics with non-specific topics was only found for two participants. We will return to possible task effects to explain this difference in Section 8.2.

## 8 Discussion

This paper examined the use of CLLD among native Romanian speakers in an L2 Italian or L2 English immersion context to test the role of L2 acquisition on native language attrition. The main findings from the Acceptability Judgment and the Written Elicitation task can be summarized as follows: the acquisition of an L2 property with similar behavior in the L1 and L2 (that are nevertheless different), but not a reduction of L1 input alone, causes attrition, as only Romanians in Italy behaved significantly differently from the Romanian monolingual control group. A further examination of language-external factors revealed that Romanians who immigrated during adolescence or in their early twenties were more susceptible to L2 transfer. In the Acceptability Judgment task, they accepted clitics in all discourse contexts where clitics are used in either their L2 Italian or their L1 Romanian and in the Written Elicitation task they applied clitics less deterministically. Importantly, however, participants with an earlier age of immigration typically also reported higher levels of L2 proficiency and L2 language use, which may have been the causing factor of attrition.

### 8.1 Effects of age of onset and quality and quantity of exposure

In this section, I briefly elaborate on whether someone’s language is maintained or attrited depends on a number of variables that are independent from maturational effects, such as the quantity and quality of input and the level of engagement with both the L1 and the L2. I furthermore discuss specific characteristics of the Romanian community in Italy and the group of subjects who participated in this study to discuss why language maintenance was fairly high in this study.

One question is why attrition effects tend to be higher in speakers who arrive in the L2 country during adolescence. It has been argued that teens are typically at an age where many start post-secondary education in the L2 and form social relationships outside of their heritage community. They have broader friend circles and use social media and social networking more, increasing the quality and quantity of L2 input, which in turn increases L2 proficiency and language use [Anderson and Jiang, 2018; Roehl and Stewart, 2018, as cited in Miller and Rothman (2020), who also found a difference between participants who migrated before and after the age of 22]. In consequence, they may be using the L2 to a greater extent than the L1 and to a greater extent than speakers who immigrated later in life. Examining potential effects of schooling in the L2 in our participant pool, it is noteworthy that eight out of 15 participants who arrived before the age of 20 reported high school as their highest level of education. Four of them arrived between the ages of 18 and 20 and therefore never received schooling in Italian while the other four did receive some schooling in Italian (one participant moved at age 14, two at age 16 and one at age 17). In other words, not all participants with an AoA during adolescence have necessarily received schooling in their L2. Additionally, the number of participants who reported high school as their highest level of education is quite high (16 out of the total 37 Romanians in Italy) and therefore many of our participants did not receive any post-secondary training in Italian either. Furthermore, most of these 16 participants are currently working as housewives, drivers or seasonal workers, jobs that likely do not require a near-native level of proficiency in Italian. When the need to speak the L2 is low, L2 interference is naturally low as well.

Additionally, rates of attrition are likely low for our participants as there are many opportunities to speak Romanian, both inside and outside their homes, and relatively few of our participants became dominant speakers of Italian. Maintaining close contacts with the L1 speaking community reinforces the L1 language system. The Romanian diaspora is the fifth largest in the world and one third of all Romanian emigrants (over 1 million) are living in Italy (OECD, 2019), a country with 55 million citizens. Most Romanians in Italy are residing in metropolitan cities such as Rome, Turin and Milan and the industrial areas of northern Italy, of which the province of Veneto is one of the largest (Stocchiero, 2002). The majority of participants in our study are family friends or members of the church community of one of the Research Assistants, who all reside in Veneto. This province has around 126,344 Romanian-born citizens (ISTAT, 2023). Our participants are part of a community of Romanian speakers with relatively high enclosure and likely have ample opportunity to maintain their native language. Many Romanians are able to successfully emigrate because of family members already residing in Italy, helping newcomers with accommodation and employment (Stocchiero, 2002). In fact, the 60 Romanians tested in our study almost all report that they speak Romanian daily (with four participants stating they speak Romanian only weekly). Additionally, given the geographical closeness between Romania and Italy, Romanians in Italy typically have ample opportunities to visit their home country. In fact, 25 out of 37 participants in Italy report to visit Romania at least once or twice per year. Furthermore, all but two participants in this group report to frequently be in touch with Romanian relatives and friends in Romania, with whom they exclusively speak in Romanian. Remarkably, the left plot in Figure 7 also showed that about half of our participants use Romanian on a daily basis at least as much as they use Italian, despite living in Italy.

Even though language maintenance levels are high for the speakers of the Romanian community in Italy with whom we conducted the experiments, we do find effects of L2 induced attrition and clear cases of individuals in our study for whom a shift in language dominance to the L2 caused restructuring of the L1 grammar. For example, two of our participants who immigrated at the ages of 11 and 12 showed complete L2 convergence in the non-specific topic condition. These individuals immigrated during high school age and obtained a university degree in Italy immigrated during high school age and obtained a university degree in Italy. They are now working in an Italian-speaking environment and use Romanian mainly with their families. These findings are in line with previous studies that showed that speakers who use their L1 mainly in informal contexts experienced higher attrition levels (Schmid, 2007; De Leeuw et al., 2010; Schmid and Dusseldorp, 2010; de Leeuw et al., 2012; Yilmaz and Schmid, 2012). Our findings also show that individuals who arrived before their early twenties and who have become dominant speakers of the L2 show the greatest extent of non-monolingual-like use of CLLD. Experimental research on attrition naturally tends to focus on diaspora communities for which a sufficient number of participants can be found. However, these speakers naturally may have more opportunity for language maintenance, leading researchers to conclude that attrition levels are low. This observation encourages future attrition research to keep the L2 community status into consideration as a factor contributing to L1 attrition.

### 8.2 Grammatical attrition and task effects

In Section 2 we discussed the question whether performance differences between attriters and non-attriters are the result of a conflict between the two grammars at the level of sentence processing alone or whether they constitute a difference in knowledge representation. It is relevant to observe that both approaches discuss the notion of “optionality,” where attriters accept both an interpretation/structure allowed in the L1 as well as the equivalent from the L2. This is in fact the most attested form of attrition for morpho-syntactic phenomena (as opposed to only using/accepting the L2 option). For processing theories like the Interface Hypothesis (Sorace, 2011), optionality means that L1 attriters are less deterministic in their choices. For the Attrition via Acquisition model (Hicks and Domínguez, 2020), a theory of linguistic representation, optionality between L1-L2 structures suggests that both options are part of the grammar and can therefore be grammatical for an attrited speaker. Since all experiments are measures of performance, it is often hard to disentangle which differences between attriters and control groups represent a qualitatively different grammar and which are temporary effects of crosslinguistic influence from the L2 grammar. For the results of the current study, however, I argue that the reported findings indicate a clear modification of the L1 grammar. Namely, for the conditions where the L1 and the L2 differ, which is with non-specific topics and with specific foci, one may expect attriters to accept both clitic and no-clitic sentences, as each can be parsed by either one of the options available in the L1 or the L2. For left dislocation with non-specific topics, for example, Italian does use a clitic but Romanian does not, and an attriter who has both options available is then expected to accept both the clitic and the no-clitic sentences. However, speakers in the attrited group (the L2 Italian speakers in Group 1) rate clitic sentences as more acceptable than no-clitic sentences, suggesting that the L2 grammar replaced the L1 options for that particular condition. The additional option of CLLD in Italian was added to the L1 Romanian grammar of attrited speakers and within that specific context the option of adding a clitic replaced the availability of no-clitic sentences.

One surprising finding is the fact that we did not find an increased use of clitics with non-specific topics in the Written Elicitation task for more speakers who rated clitics as acceptable in this condition in the Acceptability Judgment task. This difference in outcome may be due to the difference in task demand. While no time constraint was applied to either of the offline tasks, the Acceptability Judgment task naturally requires participants to respond faster than the Written Elicitation task. Since participants cannot hear the question-and-answer pair again, this task more naturally taps into the speaker’s intuition. The Written Elicitation task is more meta-linguistic in nature and participants may have applied formal reasoning strategies when giving their responses, if they become aware that clitics are used only with specific objects (shown in the experiment with definite articles). As linguists we use the intuitive judgments of speakers to describe their mental grammars. Since the Acceptability Judgment task more clearly taps into intuitive knowledge, the results from this task may be a better representation of the speakers’ grammars. In future studies, it is important to include other tasks, such as a spoken elicitation task, where participants cannot apply formal reasoning strategies due to communication pressure, or a self-paced reading experiment to measure in which discourse contexts participants expect a clitic.

### 8.3 Connecting L1 attrition to L2 acquisition

Research on L1 attrition has started to become more systematically connected to research on L2 acquisition and resemblances have been observed between the performances of near-native speakers and attriters (see for example Sorace, 1999, 2000a, b, 2003 on the use of overt pronouns in pro-drop languages). Montrul (2020, p. 214) furthermore states that “Fossilization … could be seen as the opposite of attrition because despite optimal input, the inference module seems not to be engaged or fails to become engaged to change grammatical representations.” More clearly, what fossilization and attrition have in common is that the perceptual representations of the grammars in the minds of speakers are not compatible with the input these learners are exposed to. A crucial difference between L2 end-state grammars and L1 attrited grammars is that while few L2ers reach full target-like competence in the L2, L1 grammars rarely change. Hicks and Domínguez (2020) explain this so-called paradox by suggesting that speakers continue to process input for the purpose of acquisition, as long as there is some new form of input that is structurally different from the existing mental L1 grammar. In the case of CLLD, there is coactivation with a competing L2 system due to the structural overlap between Romanian and Italian.

Since the Attrition via Acquisition model characterizes attrition as a potential outcome of acquiring another language, attrition is predicted to be possible only when L2 acquisition has occurred. The current study did not examine the L2 grammars of Romanians in Italy. This is, however, the exact population tested in Smeets (2023), who reports on the results of English and Romanian L2 speakers of Italian and English and Italian L2 speakers of Romanian at two levels of proficiency: high intermediate/advanced and near-native. The study used the same Acceptability Judgment and Written Elicitation task and only the results from the Italian-Romanian speakers are relevant here, as no transfer is possible for L1 English groups. The acquisition task involved the reorganization of grammatical features from the transferred L1 grammar to match those of the L2 input. Specifically, for complete acquisition, Romanian L2 learners of Italian have to remove the [+specific] feature and acquire the [+anaphor] feature, which proves to be successful if they start using clitics with fronted non-specific topics and stop using clitics with specific foci. Italian L2 learners of Romanian have to do the opposite by removing the [+anaphor] feature and replacing it with a [+specific] feature. The results from both tasks showed that at the near-native stages of L2 proficiency, but not earlier, speakers in both learning directions were able to broaden the contexts that use a clitic in the L2 (grammatical expansion), but L1 pre-emption difficulties were attested as well. That is, Italian L2 learners of Romanian correctly acquired the use of clitics with fronted specific foci and Romanian L2 learners of Italian did so with fronted non-specific topics. However, neither group rejected or stopped using clitics in discourses where they are allowed in the L1 but not in the L2: Romanian L2 learners of Italian continued to use clitics with specific foci and Italian L2 learners of Romanian used clitics with non-specific topics. Thus, the study found that the L2 options were added to the options transferred from the L1. Like the first-generation immigrants in the current study, most near-native L2ers in Smeets (2023) had been acquiring Italian (or Romanian) for 10+ years and had been living in Italy (or Romania). Their L2 grammars had likely fossilized.

Similar findings are reported in Gürel (2002, 2007), the first study to directly compare the knowledge representation of L1 attriters to L2 near-native speakers, which also found that in both end-state and attrited grammars, the options from the L1 and the L2 are merged. She compared the Turkish of Turkish speakers living in North America (end-state L2 English speakers) discussed in Section 2 to the Turkish of English speakers living in Turkey (end-state L2 Turkish speakers) on their interpretation of the binding properties of the Turkish pronoun *o.* Recall that English pronouns *him/her* can be bound within a larger domain in English than Turkish *o*. Gürel (2002, 2007) ‘Set-Theoretic Language Attrition Model’ suggests that attrition is most favorable when the L2 allows a superset of the interpretations available in the L1 and therefore when the L1 is more restrictive. The results indeed showed that both the attriters as well as the near-native speakers of Turkish added the interpretation from English pronouns *him/her* to that of their Turkish grammars. In other words, the L1 affected near-native L2 grammars similarly to how L2 grammar affects L1 attrited grammars. In both L2 acquisition and L1 attrition, the input data from one of the grammars provides evidence for this additional option. Note furthermore that the premise of the AvA is similar to that of the ‘Set-Theoretic Language Attrition Model’, as it suggests that grammatical attrition is disfavoured if it involves losing an option from the L1. In fact, Hicks et al. (2024) recently found no attrition in Spanish immigrants to the United Kingdom on their aspectual interpretations of the Spanish present tense and attribute this to the fact that while Spanish allows both an ongoing and habitual interpretation, English only allows the habitual interpretation. Exposure to L2 English can therefore not add options to the L1 grammar that do not already exist in the L1. In the current study we also found that Romanians in Italy can add the use of clitics with non-specific topics, as they are also used in this discourse context in Italian, but attrition does not involve the loss of clitics with specific foci.

## 9 Conclusion

This study examined whether the interpretational properties of Clitic Left Dislocation in Romanian first generation immigrants are subject to grammatical attrition. We discussed that continued activation of a similar property in the L2 grammar can impact parsing strategies of the L1 to an extent that the L2 parse can (permanently) change the grammatical representation of that syntactic construction. We focused on a discourse-syntactic phenomenon called Clitic Left Dislocation and examined whether grammatical attrition, in the form of L2 options or morpho-syntactic properties being added to the L1 grammar, can occur in the L1 linguistic competence of native Romanian speakers who are late L2 learners of either English or Italian. Results from the Acceptability Judgment task most specifically showed attrition in Romanian native speakers who moved to Italy during adolescence and who were likely most integrated in the L2 Italian community. While Romanian L2 speakers of English and Romanian immigrants to Italy who moved after adolescence did not differ from Romanians in Romania, earlier immigrants to Italy allowed clitics in Romanian also in the discourses where they are allowed in Italian but not in Romanian. Our findings contribute to an increasing body of literature showing that L1 attriters and L2 learners can end up with very similar grammars and confirm the importance of studying second language acquisition and L1 loss within a broader picture of bilingual development.

In keeping with the research topics “Experimental Approaches to the Acquisition of Information Structure” the current study drew on linguistic data from underrepresented populations, as CLLD has not previously been studied in attrition research, as well as underrepresented languages, as the L2 acquisition of discourse constraints on CLLD has previously only been studied using English native speakers acquiring an L2 with CLLD, mainly Spanish. We furthermore examined the research subjects from different methodological perspectives.

## Data availability statement

The datasets presented in this study can be found in online repositories. The names of the repository/repositories and accession number(s) can be found at: https://osf.io/5xmfw/?view_only=3c03f17e284b409fbc328316d3c150c0.

## Ethics statement

The studies involving humans were approved by Office of Research Ethics, York University. The studies were conducted in accordance with the local legislation and institutional requirements. The participants provided their written informed consent to participate in this study. Written informed consent was obtained from the individual(s) for the publication of any potentially identifiable images or data included in this article.

## Author contributions

LS: Writing – review & editing, Writing – original draft, Visualization, Validation, Supervision, Software, Resources, Project administration, Methodology, Investigation, Funding acquisition, Formal analysis, Data curation, Conceptualization.

## Footnotes

^1^Focus fronting is used most commonly to express corrective focus whereby the object is placed in a position of prominence with the goal to provide a correction.

^2^Interestingly, Pablo, the native Portuguese speaker in Chile studied in Iverson (2012) did show a compete loss of L1 forms. His case is, however, quite unique, as the speaker completely stopped using his L1.

^3^ https://osf.io/5xmfw

^4^I would like to thank Bradley Hoot for his suggestion to visualize potential effects of AoA this way.
